# Improve your success with fish cell lines—small things that matter

**DOI:** 10.1007/s11626-025-01042-1

**Published:** 2025-04-09

**Authors:** Anita Solhaug, Georgina C. Dowd, Vivian R. Dayeh, Hilde Sindre, Lucy E. J. Lee, Niels C. Bols

**Affiliations:** 1 Norwegian Veterinary Institute, 1433 Ås, Norway; 2 The New Zealand Institute for Plant and Food Research Limited, 293 Akersten Street, Nelson, 7010 New Zealand; 3 Department of Biology, University of Waterloo, Waterloo, ON N2L 3G1 Canada; 4 Faculty of Science, University of the Fraser Valley, Abbotsford, BC V2S 7M8 Canada

**Keywords:** Fish cell culture, Primary culture, Cell line development, Invitromatics, In vitro toxicology

## Abstract

There is a drive towards reducing animal experiments and developing robust biologically relevant in vitro models based on cell lines, including those derived from fish. At the time of writing, Cellosaurus, the knowledge base of current cell lines used in research, listed more than 900 fish cell lines in its database. One of the key challenges facing fish cell biology is the lack of fundamental technical information regarding the isolation, culture, and application of cell lines. Researchers often work in silos, encountering similar technical challenges, each spending significant time and resources overcoming the same issues for which solutions may not be readily accessible. Here, we share some of the key considerations for the isolation, culture, maintenance, and application of fish cell lines in toxicology, which we have encountered over our collective decades of experience.

## Introduction

**Figure 1.**
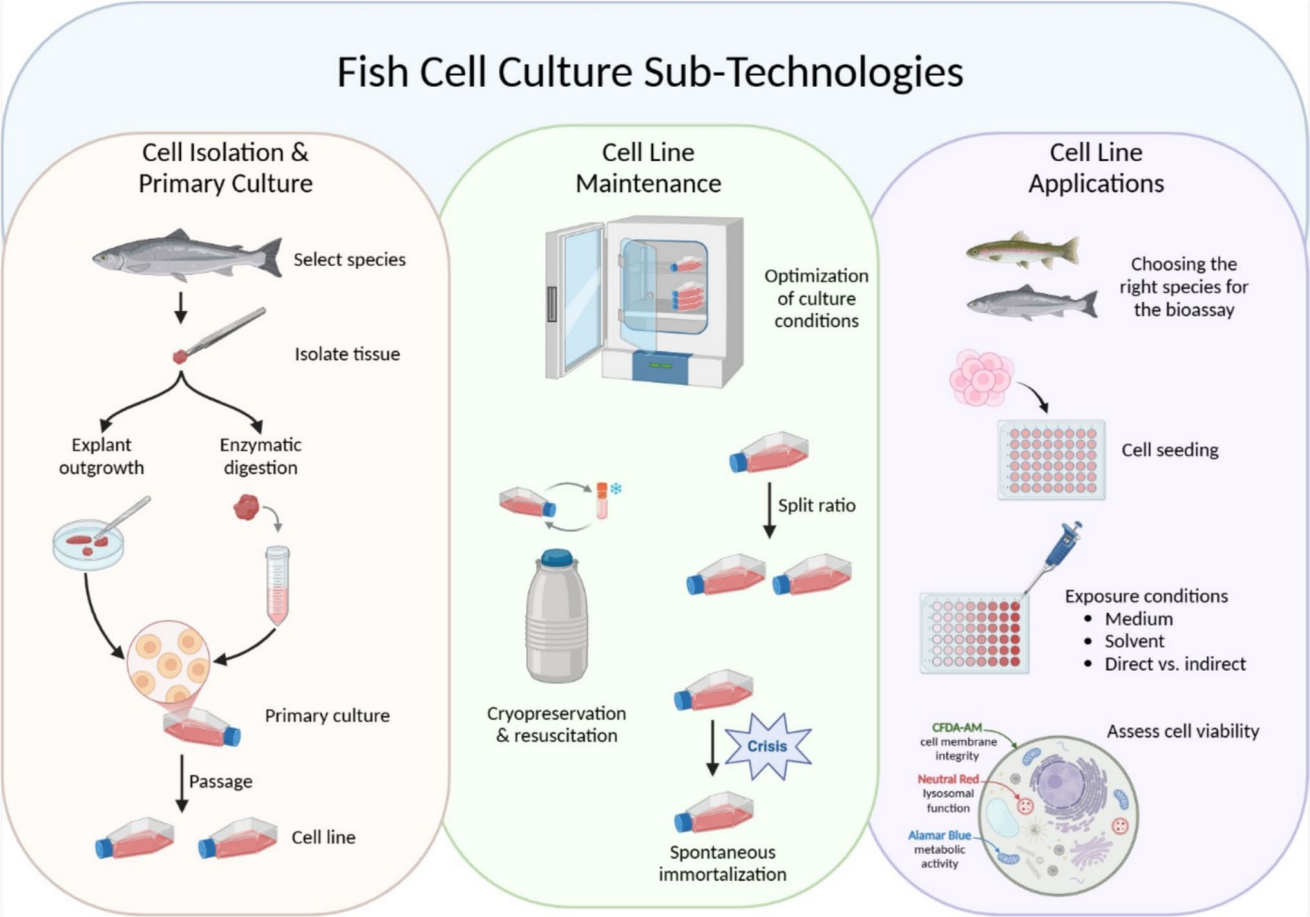
Key considerations for the development of fish cell lines and their application in toxicology. Figure created in BioRender.com.

Invitromatics can be considered a series of procedures or sub-technologies that allow cells in tissue/organ samples from multicellular animals to be grown in vitro to allow cells to be cryopreserved and made available as cell lines (Bols *et al*. 2017). The most success has been achieved for humans and laboratory rodents, with the online database Cellosaurus (Bairoch 2018), listing over 100,000 cell lines (https://www.cellosaurus.org/), which is termed the mammalian invitrome (Bols *et al*. 2017). A smaller number of cell lines have been developed from ray-finned fishes (Actinopterygii), which for convenience is referred to as the fish invitrome (Bols *et al*. 2023). Today, there are approximately 900 cell lines derived from ray-finned fishes, mostly from teleosts or Teleostei and a few from two groups more basal in the phylogeny of ray-finned fish, the Chondrostei and Holostei (Bols *et al*. 2023). Figure 1 highlights the sequence of a few key benchmark steps in invitromatics, with the preparation of a primary cell culture setting the process in motion. A cell line begins with the successful sub-cultivation or passaging of the primary cell culture and is established with the continued growth and passaging of subsequent cell cultures to yield a population large enough to allow some cells to be cryopreserved.

To date, the media, reagents, and procedures that have been developed for mammals have mostly also worked for fish (Wolf and Quimby 1962; Bols and Lee 1994; Cardoso *et al*. 2024) but fish cell lines have been reported for only between 1 and 2% of the approximately 35,000 Actinopterygian species (Bols *et al*. 2023). The abiotic requirements have been simple: cultures are always kept at 1 atmosphere of pressure and usually in an atmosphere of air at room temperature (Bols *et al*. 1992). Yet, a few Actinopterygians live in extreme aquatic environments, such as exceptionally low (Eastman 2005) or high (Pacher *et al*. 2024b) temperatures or at great ocean depths (Haedrich 1996), and the abiotic culture conditions for cells of these rare species would likely be different and unique. By contrast, the biotic factors for different ray-finned fishes appear similar and comparable to those for mammals. For example, explant outgrowth in the basal medium, Leibovitz’s L- 15, which was developed for mammalian cells (Leibovitz 1963), supplemented with foetal bovine serum (FBS), which evolved to support bovine foetal cells, permitted the development of cell lines from a sturgeon (Chondrostei) (Vo *et al*. 2018) and a gar (Holostei) (Liu *et al*. 2019), as well as numerous members of the Teleostei, with rainbow trout and walleye being just two examples (Vo *et al*. 2015b).

Despite the broad strokes of fish invitromatics being well known, our experience of several decades in developing fish cell lines and using them in toxicology has been that the key to success often can be attributed to the careful execution of a single or a multitude of sub-technologies (Segner 1998; Bols *et al*. 2005; Schirmer 2006; Lee *et al*. 2009; Dayeh *et al*. 2013; Tan and Schirmer 2017; Sindre *et al*. 2021; Slattery *et al*. 2023; Böhmert *et al*. 2024; Nguyen *et al*. 2024; Fon *et al*. 2025). Although appearing small, and in some cases anecdotal, these procedures are critical to the overall success of the discipline, and hopefully, by documenting some of them here they will be helpful to other researchers. In this article, technological details that have received less attention in other more comprehensive reviews are presented in two sections. In the section “Technical considerations for cell isolation, primary culture, and cell line maintenance”, the attention is on particulars in developing and maintaining fish cell lines, rather than lists of cell lines, which have been thoroughly documented elsewhere (Thangaraj *et al*. 2021). In the section “Technical considerations for application of fish cell lines, focusing on toxicology studies”, the focus is on aspects of using fish cell lines successfully in toxicology and/or ecotoxicology, rather than in the many other potential uses, such as in virology (Pham *et al*. 2020).

## Technical considerations for cell isolation, primary culture, and cell line maintenance

Many technical considerations for isolating and establishing primary cell cultures also apply to routine maintenance or cell cultures. Some of these considerations are discussed below.

### Tissue dissociation for cell line initiation

In general, fish cell line initiation typically involves three main dissociation methods: mechanical disruption, chemical digestion, and enzymatic digestion. These methods can be applied either individually or in combination.

Mechanical disruption involves breaking down the tissue of interest into smaller fragments by mincing it with a sharp sterile scissor or scalpel. The choice of scalpel blade (e.g. curved or triangular) depends on user preference, but it is essential to use blades that are sterile and sharp to minimise contamination and reduce cell damage, ensuring clean cuts rather than dragging with a dull blade. Mincing with scalpels is particularly useful for fibrous or dense tissues, such as skeletal muscle, gill, fin, and skin where a more forceful dissociation is needed (Fernandez *et al*. 1993; Chong *et al*. 2022; Böhmert *et al*. 2024; Xue *et al*. 2024). In contrast, cell strainers are beneficial for softer tissues like spleen and brain, where gentler dissociation minimizes shear stress and preserves cell viability (Harada *et al*. 2022). Methods using cell strainers have been applied in fish for generating single-cell suspension cultures from zebrafish embryos and *Acipenser baerii* (Siberian sturgeon) (Ryu *et al*. 2016; Samsa *et al*. 2016).

Fragments from mechanical dissociation can be placed directly into culture vessels such as multiwell plates or 12.5-cm^2^ cell culture flasks to promote outgrowth of cells from the tissue pieces, a process known as explant outgrowth/culture. For optimal results, explants should be cultured in a low volume of cell culture medium to encourage attachment. Additional medium can be added once emerging cells can be visualised. Care is crucial when handling plates or flasks with explant cultures, as sudden movements can dislodge tissue fragments, disrupting cell attachment and growth. Mechanical digestion is often combined with enzymatic digestion to improve tissue dissociation. Commonly used enzymes include collagenase, trypsin, dispase, and proteinase K. The effectiveness of these enzymes depends on several factors such as the length of incubation, the size of the initial tissue pieces, and the type of tissue being processed. For example, we find that muscle tissue typically requires a longer digestion time than liver tissue. To optimise results, we recommend using temperature ranges that mirror the host fish’s natural environment rather than the enzyme’s optimal temperature. Additionally, care should be taken to ensure the active enzyme units remain consistent, as activity can vary between enzyme batches. Enzymatic digestion can also be combined with chemical methods such as treating cells with a combination of trypsin and ethylenediaminetetraacetic acid (EDTA). The EDTA binds to calcium and magnesium, weakening cell–cell contacts to facilitate dissociation. When plating single cells or small cell clumps, allow 24–48 h for cell attachment before removing media containing dead cells. In recent years, a recombinant form of trypsin (TrypLE) that is an animal-free trypsin alternative has made cell disassociation more effective and convenient. Partial medium changes can be performed following tissue dissociation within the first 48 h to remove any traces of chemical or enzymatic dissociation agents. As with explant cultures, handle the culture flask gently to avoid dislodging cells and disrupting their attachment.

### Cell selection

In fish, selection of a specific cell type (e.g. epithelial, endothelial) during isolation or culture is relatively unexplored in comparison to mammalian cell culture where cell sorting via flow cytometry and magnetic activated cell sorting is routine. These methods are largely unused in fish cell biology due to their chemistry which requires specific antibodies, many of which are not available for various fish species.

The majority of fish cell lines developed to date have been initiated through explant outgrowth (the section “Tissue dissociation for cell line initiation”) or culture of individual cells following complete digestion of the tissue. Primary cultures established in this way usually lead to a heterogeneous cell mix. Through various selection processes which are not always clearly defined or selected for a particular reason, a single cell type (“survival of the fittest” model) or a heterogeneous mix of two or more cell types (“co-dependent cell model”) will result (Bols *et al*. 2023). Over time, these heterogeneous mixes may result in a single cell type through active or passive/accidental selection. Active selection may be achieved through adjusting culture conditions such as culturing cells in the presence of a specific growth factor (Chong *et al*. 2022), limited dilution methods (Friesen *et al*. 2025), or cell sorting techniques. Widely used in mammalian systems, fluorescence-activated cell sorting (FACS) and magnetic activated cell sorting (MACS) have been used to isolate specific cell types from heterogeneous populations, including stem cells, immune cells, and epithelial cells, based on distinct surface markers or other properties (Fong *et al*. 2009). Their use in fish culture is minimal due to limited availability of fish-specific antibodies, and low cross-reactivity with mammalian antibodies (Liongue and Ward 2007). Despite this, FACS has been successfully used for isolation of specialised kidney cells and neural cells from zebrafish (Bates *et al*. 2016; Di Giaimo *et al*. 2019). While specific studies detailing the use of cloning rings or cylinders or gradient centrifugation for single-cell selection in fish cell cultures are limited, these techniques are well-established in cell biology and have been applied across various species (Torreggiani *et al*. 2019; Sargiacomo and Klepinin 2024).

It is well known that lower vertebrates have a higher number of adult stem cells in their tissues and drive continuous growth; there is a possibility that fish cell lines are derived from the more numerous adult stem cells within tissues, while differentiated cells eventually die off (Stolper *et al*. 2019). For example, RTL-W1 cell line (Lee *et al*. 1993) was ultimately shown to have derived from liver oval cells (adult liver stem cells) from rainbow trout liver (Malhão *et al*. 2013).

### Medium selection and supplementation

Medium formulations play a significant role in the growth and survival of cells in primary culture and during routine maintenance. Fish cells are typically cultured at room temperature and often outside of a CO_2_ incubator. As a result, these cultures are exposed to ambient air rather than a controlled atmosphere of 5% CO_2_ with 95% air. To accommodate this, the buffering system in the media must differ from the bicarbonate-based systems commonly used in mammalian cell culture. One frequently used medium is Leibovitz’s L-15, which maintains pH stability by incorporating galactose instead of glucose and high concentrations of basic amino acids (Leibovitz 1963; Bols and Lee 1994). Other commonly used media include Medium 199 and Dulbecco’s modified Eagle medium (DMEM), which are typically self-buffering containing Hanks’ Balanced Salt Solution (HBSS) or HEPES buffer. While these media are standard, alternative medium formulations and use of CO_2_-incubators can be explored, particularly during the initial isolation phase. Fish cells are routinely cultured in the dark which avoids light-induced deterioration of media (Neutsch *et al*. 2018). Evidence suggests that medium requirements can vary not only between species but also among different cell types within the same species (Chong *et al*. 2022; Böhmert *et al*. 2024). Other important considerations are the type and concentration of serum in the media. A basal medium is almost universally supplemented with FBS to make a complete medium for the growth of a fish cell line. Attempts have largely failed with serum from other bovine life stages, newborn calf serum (NCS) and calf serum (CS). The Chinook salmon embryo cell line (CHSE- 214) died in NCS and showed little or no proliferation in CS (Ganassin and Bols 1992). Sera from other species have also been generally ineffective. Sera from salmon (Fryer *et al*. 1965; Collodi and Barnes 1990) and from horse and pig (Ganassin and Bols 1992) were toxic to CHSE- 214 cells or failed to sustain their proliferation. On the other hand, goat serum supported in primary cultures the attachment and proliferation of cells from the Indian major carp (Nanda *et al*. 2009). Although FBS is by far the most common supplement and usually added to basal media to give 10% (v/v), other FBS concentrations have been used to favour particular cell types. For example, high (20–30%) FBS was critical to the development of the spleen monocyte/macrophage cell line, RTS11, but once developed RTS11 could be maintained at 10–15% FBS (Ganassin and Bols 1996, 1998). Lowering the concentration of FBS to 5% appeared to aid the development of RTL-W1 as an epithelial liver cell line (Lee *et al*. 1993). Higher concentrations of FBS can improve the success of cell culture, but may preferentially select for fast-growing cells, such as fibroblasts. Therefore, the choice of serum concentration should align with the intended application of the cultured cell type. Current research is being done to eliminate the use of FBS for fish cell lines, and recently, excellent progress was made in this direction with RTgill-W1 (Jozef *et al*. 2025).

Nearly all researchers add a mix of antibiotics/antimycotics to primary fish cell cultures to prevent microbial contamination but once a cell line has been developed they may be removed. The antibiotics/antimycotics and the concentrations at which they are used are the same as have classically been used with mammalian and avian cells (Kuhlmann 1995). The issue of mycoplasma usually emerges with the development of a cell line and possible actions to be taken have been prominently discussed in a relatively recent review (Bols *et al*. 2017). Mycoplasma are insidious because they cause no obvious change in phenotype but might be altering properties of the cells in unknown ways. Yet even mycoplasma-infected fish cell lines have provided valuable insights into the complex interactions between fish pathogens (Schachner *et al*. 2017). However, for nearly all other purposes, the use of mycoplasma-free cell lines is strongly desired. With antimycotics and protocols that have been used successfully with mammalian cell lines, mycoplasma have been removed from fish cell lines (Bols *et al*. 1994). Any flasks which are found to be contaminated should be disposed of immediately, ensuring that neighbouring culture vessels are not contaminated in the process.

Unlike mammalian cells, many fish-specific growth factors are not commercially available, prompting the need for alternative options. Cell secretome/conditioned media, rich in proteins including growth factors and hormones and small molecules such as ions and microvesicles, can be used effectively to support cells in primary culture and during early passages. Conditioned media from cell lines with high proliferative capacity, such as the ASG- 10 cell line (Slattery *et al*. 2023), may be particularly useful for this purpose. Since the concentration of various factors in this medium is unknown and may vary depending on the cell source, it is advisable to test a range of concentrations of this additive. Additionally, care must be taken to ensure conditioned media have been filter-sterilised to remove any viable cells and prevent inadvertent cross-contamination. An alternative source of growth factors for culturing fish cells is to use commercially available recombinant proteins derived from mammalian sequences. Evidence suggests fish cells can respond to mammalian growth factors in vitro. For example, the muscle-derived cell line CAtmus1PFR exposed to insulin like growth factor (IGF- 1 or IGF- 2) exhibited increased proliferation. However, caution is required, as addition of exogenous growth factors influences cell fate. For instance, exposure of CAtmus1PFR to transforming growth factor beta (TGFβ) induced differentiation of the cells into myofibroblasts (Chong *et al*. 2022).

### Plastic ware

Fish cells could be can be selective about the type/brand of plastic they adhere to and grow on. This would be due to the differences in surface treatments used by various companies to modify the plastic and enhance its polarity or charge. As polystyrene, widely used in cell culturing plastic, is hydrophobic and thus makes anchoring difficult, chemical modification of the plastic itself or coating with various substances is necessary (Chang and Wang 2011; Morán *et al*. 2019). There are multiple commercial cell plastic options, varying in hydrophobicity, charge, and roughness/softness, including oxygen-containing surfaces (negatively charged, e.g. Corning CellBIND) and nitrogen-containing surfaces (positively charged, e.g. Corning Primaria) which facilitate the growth of cells with lower binding capacity. Although plastic surfaces that have been prepared by the manufactures to have different properties, they have only rarely been explored to select for specific types of fish cells. One of the few examples tested the growth of a fibroblast (RTG- 2) and an epithelial (CHSE- 214) cell line on three different plastic surfaces that were prepared by a 1990 s tissue ware manufacturer Tekmat (Ashland, MA) and described as Plastek A (hydrophobic), Plastek C (positively charged), and Plastek M (negatively charged) (Barlian and Bols 1991). Unfortunately, both cell lines responded similarly: they only grew into monolayers on the negatively charged surface (Barlian and Bols 1991). However, as the mechanisms for adhesion and spread are complex, it is difficult to predict the best plastic option for a specific cell type and species. Using a couple of different alternatives for initial establishment is therefore advisable, especially for fish, as the knowledge on the preferences of the different cell types is often lacking.

Coating plates with extracellular matrix components, such as collagen, fibronectin, laminin, or collagen, can help cells attach initially. The choice of matrix can be tissue- or cell-specific, e.g. collagen is often used for fibroblasts (Reinhart and Lee 2002). A complex mixture of extracellular matrix components, like Matrigel, can also be used. Trying multiple conditions using a multiwell plate can help identify the optimal environment for cell attachment and growth.

### Culture environment and temperature

Fish cells are routinely cultured in non-humidified environments, such as closets or drawers (section “Medium selection and supplementation”) using cell culture media, like L-15, that are designed to support cell growth in environments without CO_2_ equilibration (Leibovitz 1963). Under these conditions, media evaporate at varying rates over time, leading to increased osmolarity and reduced capacity to support cells. The most effective solution is to use non-vented flasks which significantly slow evaporation. In our experience, wrapping parafilm around a vented/filter cap is not sufficient and a non-ventilated cap is essential. The relatively porous nature of cell culture flasks allows for adequate gas exchange, which is preferred by fish, as they are generally hypoxia-tolerant and thrive in low-oxygen conditions rather than hyperoxic ones (Kleeman *et al*. 1970). When using other media, such as DMEM, cells usually must be cultivated in a CO_2_ incubator (Bols and Lee 1994). Be aware that most CO_2_ incubators require temperatures above room temperature to function correctly. Although CO_2_ incubators can be modified to maintain temperatures below room temperature, the cost of modifications can be prohibitive. A less expensive option for the use of DMEM below 21–22°C is to use a simple low-temperature incubator and a DMEM that has been modified with the addition of HEPES, which allows the pH of the cell cultures to buffered without an atmosphere of CO_2_.

To date, nearly all fish cell lines appear capable of some proliferation at “room temperature”, usually between 20 and 22°C. For most existing fish cell lines, culturing at room temperature is generally not problematic. However, selecting the appropriate temperatures for fish cell line maintenance and experimentation is more nuanced than it may initially appear.

While culturing cells at room temperature by incubation in a drawer or closet is widely practised, it has both practical and perceptual drawbacks. Maintaining cells in a desk drawer, for instance, appears to lack scientific rigor or accuracy. Moreover, environmental temperature fluctuations, such as irregular heating that raises the temperature to 25°C, can stress temperature-sensitive cells like those from rainbow trout if the elevated temperatures persist for a day or more (Bols *et al*. 1992; Pumputis *et al*. 2022). For this reason, ongoing monitoring of environmental temperatures is recommended in facilities where fish cells are incubated outside of a controlled incubator. Modern buildings generally maintain temperatures within a narrow range of 20–22°C, and even dedicated incubators are subject to minor fluctuations. In our experience, more fish cell lines have been lost due to incubator-related issues—such as malfunctions after power outages or accidental temperature adjustments by researchers—than through room temperature maintenance. As such, if the thermal biology of the fish species supports some degree of cell growth at room temperature, even if it is not ideal, maintaining a few culture flasks at room temperature can serve as a practical and reliable backup, particularly during the early stages of cell line development.

A temperature-controlled incubator allows researchers to select a temperature that is optimal for the in vitro proliferation of cells from a particular fish species, although in most cases, this temperature will only approximate the species’ true thermal biology. Identifying the optimal proliferation temperature(s) is an important practical consideration, as it determines the best temperature for maintaining a cell line and producing cells for experimentation. The actual experiments can be conducted at different temperatures (Pumputis *et al*. 2022), depending on the research goals and the thermal biology of the species. Incubators generally perform best when set to a temperature slightly above or below the ambient temperature, usually room temperature. For rainbow trout cells, an incubator set at 18°C effectively provides a stable temperature for routine cell line maintenance. For many fish species, limited information is available on critical temperatures, and room temperature incubation offers a straightforward method to begin identifying these temperature ranges at the cellular level.

As efforts to establish cell cultures from extremophile fish species continue, it is possible that some cell lines may not be able to grow at room temperature. For instance, fish from Antarctic marine environments, where the water temperature remains close to the freezing point of seawater at − 1.86°C (Matschiner *et al*. 2015), may not be able to survive long enough at room temperature for cell proliferation to occur. On the other hand, some fish species can tolerate temperatures up to 40°C or slightly higher for short durations (Martínez *et al*. 2016; Pacher *et al*. 2024a). One example is the cell line PLHC- 1, derived from *Poeciliopsis lucida*, which can grow at room temperature, although its optimal growth occurs at higher temperatures around 30°C (Hightower and Renfro 1988).

### Sub-cultivation − Considerations specific to primary culture vessel

Once the primary cultures have been initiated, the concerns over the first few days and weeks are the possible emergence of microbial contamination and of cellular deterioration. For the first, the cultures should be observed with the naked eye and an inverted phase-contrast microscope. With the naked eye, the signs of extreme microbial contamination are the culture medium turning yellow, showing a change in turbidity or acquiring surface films. In these cases, the action is clear: autoclave the cultures and discard. In those instances where cultures appear fine by the naked eye but have suspicious features by phase-contrast microscopy, several actions can be recommended. The simplest one is to terminate the cultures. On the other hand, actions can be undertaken in attempt to clarify a possible problem. One is to change medium repeatedly in the hope that microbes can either be washed away completely or greatly diminished so that the antibiotics kill any remaining. An alternative action is to change the medium to one without antibiotics to see if microbes are indeed present and will make themselves obvious by turning the medium yellow. If they do, the action would be again to terminate culture. However, if nothing emerges, development of the cultures can proceed and the focus can return to the fish cells.

The primary cultures should be also monitored by phase contrast microscopy for the health of the fish cells, especially in the first few days and weeks, but subsequent actions can be variable. The first positive sign is the appearance of cells and/or cell fragments attaching and spreading out over the growth surface. As long as any concerns of possible microbial contamination have been assuaged as discussed above, several courses of action can follow and this will be illustrated by two extremes, but keep in mind an intermediate course might be best. In the first extreme, the medium would be changed frequently over the next few weeks but the vessels would be handled carefully and not roughly, which might cause cells to detach. Changing the medium removes cell debris, which could have arisen during the tissue dissociation process and/or cells dying, and if unremoved, could impede further cell attachment, spreading, and growth. Changing the medium also removes the build-up of metabolites, such as ammonia that can cause the appearance of vacuoles but with its removal the vacuoles disappear (Solhaug *et al*. 2024a; Pritchard *et al*. 2025). However, changing the medium usually does not change the appearance of large, well-spread cells, which by phase-contrast microscopy have stress fibers radiating through their cytoplasm. These cells often stain with senescence-associated β-galactosidase (Vo *et al*. 2015b) but with such staining the culture obviously can no longer be used to develop a cell line, which is the goal of this section. The extreme opposite to the frequent changing of the medium strategy is to leave the primary culture undisturbed for weeks or months and let the cells work it out (Bols *et al*. 1994). With both courses of the action, the heterogeneity of the culture will likely be changing but the goal is to build up enough cells for next step, sub-cultivation, or passaging.

Once cells in the primary culture reach 80–100% confluence, they can be gently removed from the culture surface for sub-cultivation, commonly referred to as passaging or splitting. At this stage, some researchers selectively dislodge specific cell types to establish more homogenous cultures. Examples of techniques include using selective attachment or detachment for the separation of fibroblasts from epithelial cells in the culture. This involves adding a detachment reagent, such as trypsin to the culture, for a specific duration, and then quickly transferring the first cells to dislodge from the surface into a new vessel and then performing a second sub-cultivation of the remaining cells to a separate flask. Several passages using this technique may be required to establish a more homogenous culture from one that is originally heterogeneous.

### Considerations for all culture vessels

With only a few exceptions (e.g. RTS11 and CHSE-sp in the salmonid invitrome), fish cell lines typically grow adherently. To maintain the cell line or use the cells in experiments, they must be temporarily detached from the plastic surface of culture vessels. This requires a cell detachment procedure to create a cell suspension for passaging/splitting or for seeding into experiment vessels (e.g. multiwell plates). While non-enzymatic cell-dissociation solutions have been used for CHSE- 214 (Barlian *et al*. 1993), most adherent cultures rely on enzymatic solutions for detachment. For suspension cells, sub-cultivation is achieved by repeatedly pipetting the medium to breaking up aggregates of floating cells and aliquoting the resulting cell suspension into new vessels.

Enzymatic cell detachment is typically performed using commercial enzyme preparations with trypsin or trypsin-like activity. For the first 30 years, trypsin was mostly sourced from cow or pig pancreas (Bols and Lee 1994), with cod trypsin also used successfully (Barlian *et al*. 1993). Trypsin was applied after rinsing the culture surface with Versene (EDTA) or in solutions containing both trypsin and EDTA. In recent decades, recombinant fungal trypsin-like proteases, such as TrypLE (Nestler *et al*. 2004), have become popular (Bols *et al*. 2011), as their activity is easily stopped through dilution, unlike pancreatic trypsin, which becomes inactivated with the abundance of proteins in FBS. Both types of trypsin are still in use.

Obtaining a single-cell suspension is essential for accurate counting and subsequent seeding, but on occasion, certain combinations of trypsin and cell lines can cause issues. A good example of this occurred with RTG-2 cells, where, after rinsing with EDTA, followed by trypsinisation, trituration, centrifugation, and resuspension in L-15/FBS, the cells formed floating aggregates or spheroids instead of attaching and spreading in new vessels. Even after a week, only a few aggregates were attached, with some cells spreading out from them, resulting in a sparse and uneven distribution of cells in the new culture vessels. This led to slow cell line maintenance and difficulty in setting up experimental cultures. After testing various steps, the issue was resolved by using bovine rather than porcine trypsin with no such problem observed using TrypLE.

Sub-cultivating fish cell lines using trypsin or TrypLE is generally straightforward, but performance of individual steps in the protocol can vary between laboratories and may need to be tailored for specific cell lines to generate single-cell suspensions. Key steps that can be varied include the degree of mechanical agitation, duration of enzyme treatment, centrifugation conditions, and pellet resuspension. In addition to variations in protocol steps, the sub-cultivation schedule for a particular cell line can impact the overall process. A few of these variables are discussed below. Some mechanical agitation is an inevitable part of the cell detachment process as pipetting the trypsin solution in and out helps dislodge cells. This dislodgement can be enhanced in several ways. The simplest is to repeatedly pipet the trypsin solution forcefully over the vessel surface, on which the cells are progressively rounding due to the trypsin treatment. Another is to use a “cell scraper” to help detach the rounding cells, and in some instances, a combination of gentle scraping and trypsin is necessary to release especially adherent cells, which do not completely dislodge even with prolonged incubation. For flasks, adding just enough trypsin to cover the cells, and then repeatedly slamming the bottom of the flask bottom onto the lab bench, causes the cells to pop off the surface primarily as single cells. All these methods can speed up the detachment process, but with or without them, some cell lines are more difficult to detach than others and treatment times can vary. Care must be taken to avoid excessive agitation, which could damage the cells. It is also crucial to ensure that all cells are dislodged to avoid selective loss of certain cell types during sub-cultivation.

Some researchers seed cells at a particular density, while others dilute by eye. What seeding densities or split ratios should be used really depend on the purpose of the cell culturing and to a degree on the cell line. All fish cell lines can be routinely cultured at a low split ratio (1:2 or 1:3). Such ratios are good for building up a stock of cells for use in experiments or in cryopreservation because usually within a week or two the cultures are ready to be used or subdivided further. In short, the culturist and cell line can get into a weekly rhythm. However, sometimes the purpose of sub-cultivation might be just to maintain the cell line with as little attention as possible so at some time in the future they can be a source of cells for more routine growth without having to go into the stock of cryopreserved cells. For such slow maintenance, much higher split ratios, for example, 1:10, can be used. The cells will grow much more slowly, presumably because the capacity of the culture to build up growth factors (i.e. condition the medium) is much reduced due to the low cell numbers. The advantages for the culturist are that the cells are slowly proliferating for a period when they are not needed and fewer resources, including time, are used. This strategy will not work on all cell lines and will have to be tested on a case-by-case basis. An example of a cell line that can be maintained at a high split ratio is CHSE- 214, whereas one that cannot is the walleye bulbus arteriosus, WEBA (Vo *et al*. 2015a).

### Optimising culture conditions

Once a culture has been passaged out of the primary vessel, experiments to further optimise culture conditions can be initiated, though it may take several passages before a sufficient number of cells are available for experimentation.

**Figure 2.**
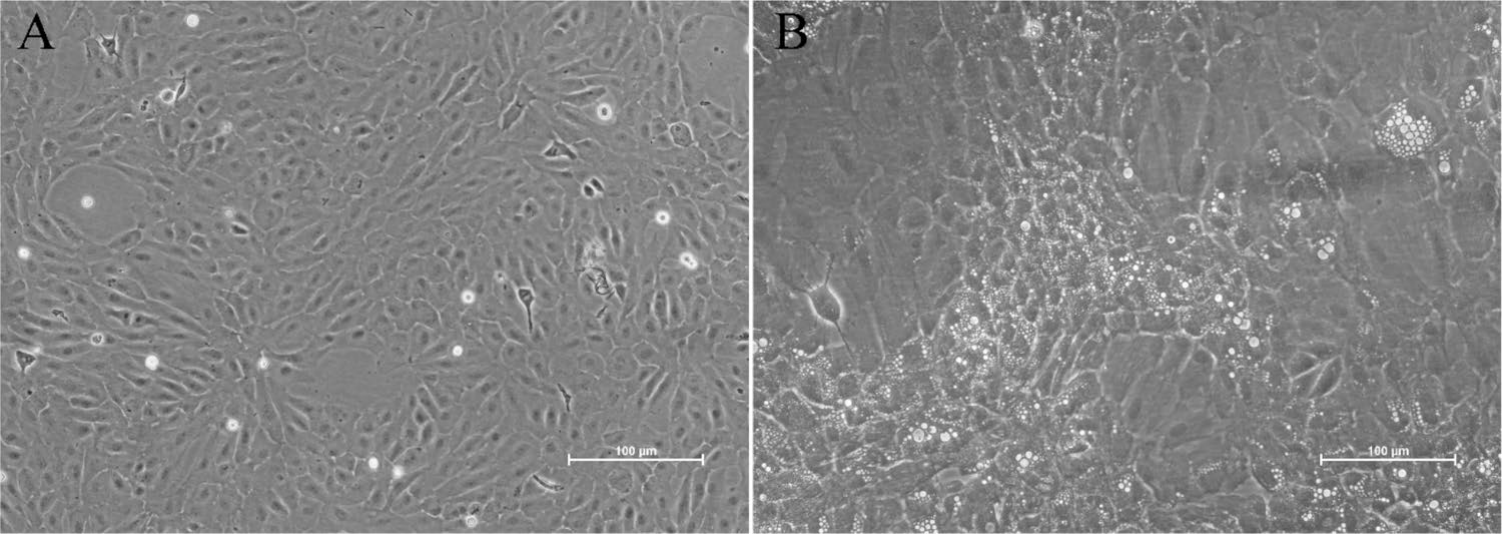
Gill epithelial cells from Atlantic salmon (ASG- 10), proliferating cells with normal morphology (*A*) and starved vacuolated cells (*B*). Photo: Anita Solhaug (Norwegian Veterinary Institute).

Similar to primary culture, adjustments to basal medium formulations (the section “Medium selection and supplementation”), serum concentration (the section “Medium selection and supplementation”), plastic ware (the section “Plastic ware”), and temperature (the section “Culture environment and temperature”) can enhance cell growth beyond the primary culture. Care should be taken to monitor cells for morphological changes, which may indicate differentiation to another cell type (which may or may not be a desired outcome) or signs of stress such as vacuolisation (Fig. 2). These types of experiments are routinely performed in multiwell plates. A key technical consideration when altering culture medium is whether the focus is on the impact of the medium on cell adherence or its effect on cell replication. This will determine whether to transition cells to the test medium during seeding or after they have attached to the culture vessel.

### Conditions for the spontaneous immortalisation of fish cell lines

Putting fish cell lines into the context of mammalian cell line concepts like senescence/crisis and immortality is challenging but offers valuable insight. Mammalian cells often face a senescent barrier after 20–60 passages, depending on cell type. Other cultures can overcome this during a transient period of crisis where a cell line emerges that is described as having arisen spontaneously. Others require directed immortalisation through factors like SV40 large T antigen and polyomavirus middle T antigen (PyMT). Fish cells, by contrast, rarely exhibit senescence and spontaneously immortalise through culture, though how many passages constitute immortality for fish cells appears to be an arbitrary decision. While directed immortalisation with PyMT has been tested in fish heart cells (Luque *et al*. 2014), the impact is difficult to gauge due to the relative ease with which fish cells naturally bypass senescence. Despite nearly all fish cell lines spontaneously immortalising (Bols *et al*. 2023), the process of achieving immortality through routine culture remains complex, as discussed below.

The progression of primary cell cultures to spontaneously immortalised cell lines is often described inconsistently, reflecting the nature of their discovery — a onetime event usually discovered after the fact. Typically, accounts are anecdotal and vague. Additionally, what constitutes immortalisation has been nebulous. In our work with salmonid cell lines, we consider cells immortalised after 20–30 passages at 1:2 or 1:3 splits and successful cryopreservation. To identify patterns, we have categorized spontaneous fish cell line development into six scenarios. Our practical advice for new fish cell culturists is to stay adaptable and patient.

1. **Cell lines from clones**. Cloning is an uncommon pathway for developing fish cell lines from post-embryonic tissues but may occur more frequently with early life-stage material (Bols *et al*. 2004; Fan *et al*. 2004). While adult-derived fish cell lines can form clones, cloning is typically observed after establishment (Chen *et al*. 2010). Examples include JEM1129, a Japanese eel cell line derived from an early passage (1–3) myoblast cultures (Ikeda *et al*. 2024), and PBLE, and an American eel cell line emerging from a clone in a year-old leukocyte culture (Dewitte-Orr *et al*. 2006). JEM1129 originated from the glass eel stage, whereas PBLE came from a non-mature adult.
2. **Crowded culture:** This method suggests that spontaneous immortalization requires adherent cells to remain in highly crowded primary or early passage cultures for some time before routine sub-cultivation and growth to confluence can begin. Typically initiated by enzymatic dissociation or explant outgrowth in flasks, these cultures are often left undisturbed for weeks to months. During this time, the cells slowly proliferate, forming tightly packed monolayers or multilayered patches. Once sub-cultivated, the cells proliferate faster, enabling regular cycles of growth, sub-cultivation (1:2 or 1:3 splits), and regrowth to confluence. This process has led to the development of multiple rainbow trout cell lines from various organs, even in mature, spawning fish (Bols *et al*. 2017, 2023). A variation of this method focuses on maintaining the original primary culture flask, which can accelerate cell line development. After sub-cultivation (the section “Sub-cultivation”), small mounds of adherent cells, consisting of tissue fragments or explants with cells yet to migrate, often remain in the flask. These cells tend to repopulate the original flask faster than those detached and plated into new flasks. As a result, the original flask can be cycled through growth, sub-cultivation, growth more quickly, becoming the source of immortal cell lines. An example of this is the Atlantic heart cell line, ASHe (Pham *et al*. 2017).
3. **Senescence or crisis:** Senescence and/or crisis temporarily disrupt cell accumulation at some point before an immortal cell line emerges. This has been observed in catfish, goldfish, and mackerel cell lines. For catfish leukocyte cultures, transitory culture deterioration occurred around 4 wk (Barker *et al*. 2000), while goldfish and Atlantic mackerel muscle cultures showed deterioration after 12–15 and 37–43 passages, respectively (Li *et al*. 2021; Saad *et al*. 2023). No standard criteria have emerged for identifying senescence or crisis in fish cell cultures. While some cell lines stain for senescence-associated β-galactosidase (SA β-Gal), others do not or show diminished staining over time (Vo *et al*. 2015b; Bloch *et al*. 2016; Futami *et al*. 2025). Crisis may also be overlooked if cultures are maintained with minimal intervention. Despite these challenges, senescence has been repeatedly induced in the classic EPC fish cell line, and the molecular mechanisms behind fish cell immortalization are beginning to be explored (Futami *et al*. 2019, 2022, 2025).
4. **Organoid-like growth**: This scenario appears to be unique to the rainbow trout gill cell line, RTgill-W1 (Bols *et al*. 1994). RTgill-W1 was developed from gill fragments that had persisted in primary culture for 16 mo, during which time the secondary lamella on primary lamellae fragments grew and lengthened. These 16-mo-old gill fragments might now be considered organoids, suggesting that organoids might provide another potential route for developing fish cell lines, warranting further attention.
5. **Long-term primary cultures:** The rainbow trout monocyte/macrophage cell line, RTS11, illustrates the potential of long-term hemopoietic spleen primary culture (Ganassin and Bols 1998). Such cultures consist of a complex adherent stromal cell layer that produces monocytes/macrophages and dendritic cells, many of which are released into the medium as single cells or cell clusters. Transferring this culture medium into new flasks without an adherent stromal layer resulted in non-proliferative cells/cell clumps in the culture which persisted in suspension for weeks to months. Eventually, one culture began to proliferate, giving rise to RTS11. Long-term hemopoietic cultures might offer a pathway for developing other blood lines.
6. **Non-dissociated tissues/fluids:** Spontaneous immortalization can occur without tissue or organ dissociation. This is apparent from the previously mentioned PBLE from eel blood and an earlier carp blood cell line (Faisal and Ahne 1990). Other biological fluids, such as the coelomic fluid and milt of spawning rainbow trout, have also been sources of cell lines (Vo *et al*. 2015b). In these cases, somatic cells present in the fluids adhered to plastic culture flasks and, after prolonged incubation, began to proliferate. Clones formed, grew into each other, and were eventually sub-cultivated into stable cell lines.

## Technical considerations for application of fish cell lines, focusing on toxicology studies

### Prepare your cells for experiments – Plating out your cells for experiments

**Figure 3.**
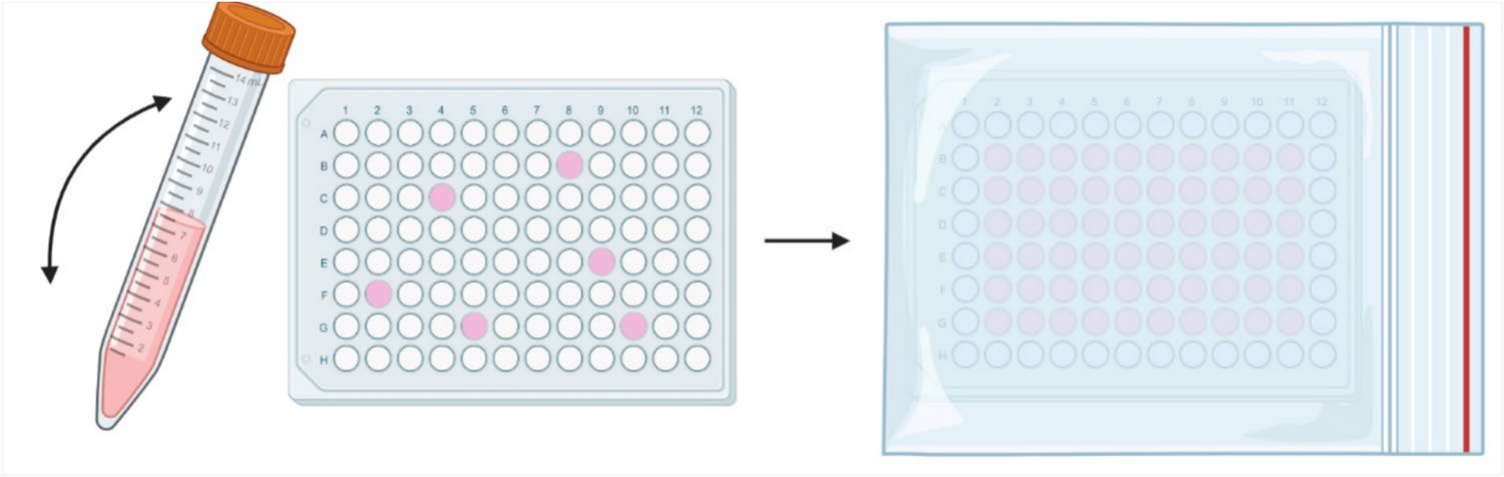
Plating out fish cells for experiments. Increasing consistency in experiments by combining frequent mixing of the cell suspension, random pipetting into wells of a multiwell plate during cell seeding, and growth to confluence in non-ventilated conditions. Figure created in BioRender.com.

To ensure experimental consistency, cells must be plated at the same cell density every time, as effects may vary with confluence. To do so, do not rely on flask cell density or split ratios — always count the cells. After determining the required density for the multiwell plate format (cells/cm^2^), prepare a cell suspension in the appropriate growth medium. Typical seeding density for multiwell plates is 132,000 cells/cm^2^, which often gives you a 100% confluent cell layer after 24 h. However, this will vary between the different cell lines due to different sizes and proliferation rates. To achieve uniform cell density across the multiwell plate, add the suspension randomly into individual wells rather than in a linear/sequential fashion (Fig. 3) and mix the cell suspension tube regularly by inverting it every 6–12 wells. This will prevent cell settling towards the bottom of the tube over time which can alter the cell density of the subsamples. For 6- to 48-well plates, gently shake the plate in a linear fashion, not circularly, to avoid cells clustering in the centre of the well. Shaking the plate is not necessary for 96-well plates. As discussed in the section “Culture environment and temperature”, fish cells often grow better under non-ventilated conditions, which can be achieved sealing the plate with parafilm or by putting the plate in a small plastic zip lock-bag (Fig. 3).

### The edge effect

The “edge effect” occurs when cells in the edge wells of a multiwell plate grow differently from those in inner wells due to variations in humidity and temperature (Lundholt *et al*. 2003; Mansoury *et al*. 2021). This can impact experimental results, particularly when detecting small responses where consistent cell numbers are critical, and the exposure reagent should be the only variable. While it is difficult to eliminate the edge effect, the following strategies can help reduce its impact:

- Fill outer wells with another solution such as PBS.
- Do not stack plates during incubation.
- Seed cells at a density where they are close to confluence.
- Use shorter exposure periods.
- Use culture medium pre-warmed to the incubation temperature for seeding.
- Allow cells to adhere to the plastic before incubating the plate.
- Be aware of incubator fans that may cause a temperature gradient over the plate.

Before starting, test for potential edge effects by plating cells at the desired density and growth conditions (without test chemicals), performing an endpoint assay (such as Alamar Blue), and analysing differences across the whole plate. Based on experience, using 48-well plates with 500 µL/well of cell culture medium is effective for minimising edge effects.

### Exposure – The exposure compound

**Concentration**: “The dose makes the poison”, a concept introduced by Paracelsus in the 1500 s, underscores the importance of evaluating the relevance of the experimental concentrations. While final concentrations may be close to those that are environmentally relevant, some toxicants, like microplastics, may require significantly higher concentrations to elicit detectable cellular responses, as environmentally relevant concentrations may result in less than one particle per well (Solhaug *et al*. 2024b). Typical exposure durations are 24 h, though shorter and longer exposure times may sometimes be more appropriate. Longer exposure times should be carefully monitored to ensure that impact on cell viability in control cultures is not due to time-related factors. Medium change during an ongoing experiment is generally not advisable, as this can lead to uncertainty in toxicant concentrations due to accumulation in the cells.

**Biotransformation**: Some cell lines can biotransform toxicants if they retain xenobiotic metabolism. Several cell lines derived from organs that perform xenobiotic metabolism, such as liver cell line RTL-W1, gut cell line RTGutGC, and the gill cell lines ASG-10, LG-1, OTgill1PFR and CAgill1PFR, have been shown to maintain activity of several biotransformation enzymes (Stadnicka-Michalak *et al*. 2018; Sindre *et al*. 2021; Ivanova *et al*. 2023; Böhmert *et al*. 2024). The metabolites produced may have altered toxicity, either increasing or decreasing effects on cell viability. To account for this possibility, biotransformation capabilities can be investigated using the EROD assay (CYP1A activity), or more precisely, by identifying metabolites produced by specific biotransformation enzymes by high-resolution mass spectroscopy (Ivanova *et al*. 2023).

**Bioavailability**: The choice of exposure medium is critical, as it can significantly impact experimental outcomes. Commonly used media include growth medium (e.g. Leibovitz’s L-15) with or without serum, typically FBS, and isotonic solutions such as L-15/ex. L-15/ex contains salts, pyruvate, and galactose like L-15 medium, but excludes amino acids. The lack of protective antioxidants and vitamins in L- 15/ex may increase cell sensitivity toward some toxicants (Dayeh *et al*. 2002). Also, increasing the salt concentration of L-15/ex can reduce this sensitivity by altering the contaminant bioavailability, particularly for metals (Minghetti and Schirmer 2016). Additionally, by adjusting the L-15/ex osmolarity towards hypo-osmotic (150 mOsmol/L), conditions have been shown to heighten sensitivity to cadmium and nickel (Scott *et al*. 2022).

Understanding the chemical properties of a toxicant, especially its lipophilic/hydrophilic properties, is key to understanding its bioavailability during exposure. Lipophilic compounds may need specific amounts of serum and/or solvents (e.g. dimethyl sulfoxide (DMSO), methanol (MeOH), or ethanol (EtOH)) for dissolution in media and cellular uptake. Additionally, serum in the exposure medium can coat the plastic surfaces, reducing the amount of the toxicant that can stick to that surface. Lipophilic compounds may accumulate in cells, making repeated exposures unreliable due to uncertain final concentrations. To confirm the exact toxicant concentration, measurement by using mass spectrometry at the end of exposure is recommended.

Efflux pumps/transport proteins in cells can also affect the bioavailability of toxicants or other chemicals. These active transporters expel unwanted materials and are highly expressed in barrier cell types, such as in the intestine, blood–brain barrier, and gills (Ferreira *et al*. 2014; Luckenbach *et al*. 2014). Several dyes, including the nuclei stain Hoechst 33342 and the ROS-probe H_2_DCFDA-AM, may be actively transported out of the cell by efflux pumps (Neuberger and van Veen 2015; Sieprath *et al*. 2017). Also toxicants, such as the algal toxin microcystin-LR (glutathione-conjugated microcyctine-LR) (Bieczynski *et al*. 2014), can be substrates of efflux pumps. When such toxicants are used, efflux pumps can reduce their bioavailability, potentially underestimating cellular effects. Variability in transporter protein expression between cells can complicate comparisons, potentially leading to confusing results (Solhaug *et al*. 2022). To overcome this effect, efflux pump inhibitors can be used. However, care should be taken when introducing inhibitors in a system, as additional unspecific effects may occur.

**Autofluorescence**: Toxicants, especially organic compounds containing conjugated double bonds or aromatic rings, are more likely to autofluorescence, usually in the blue end of the emitting spectrum. This will then give false positive results in the bioassay if the endpoint measurement is fluorescent in the same emitting area. In addition, the cells may also change/differentiate after toxicant exposure, and thus themselves represent a source of autofluorescence that may give a false positive result. To avoid this, it is important always to include a control with the test toxicant alone (Section “Assay controls”) but without any staining, to determine if there is any autofluorescence. Furthermore, it should be noted that both FBS and phenol red, common supplements in cell culture medium, are known to increase the background levels observed in fluorescence measurements. If using a plate reader to evaluate experimental endpoints, a bottom-reader can give reduced autofluorescence from cell culture medium compared to a top-reader. A low background (high signal-to-blank ratio) is crucial for a low detection level. Furthermore, if autofluorescence from your toxicant is detected, you may use a fluorophore for detection that emits in the far-red spectrum or select another readout such as luminescence.

### Choose the right solvent carrier

Common solvents for hydrophilic compounds include water, PBS, or a cell culture medium. These will have no or only minor effects on the cells as long as they do not affect the ionic balance or highly dilute your cell culture medium.

Both MeOH and EtOH are suitable carrier solvents for lipophilic compounds. However, these solvents evaporate and are much more difficult to pipette in such small volumes; and thus, a positive displacement pipette is recommended. However, since these solvents easily evaporate, they are compatible with analytical chemistry in contrast to DMSO. At the cellular level, both MeOH and EtOH increase the fluidity of membranes, where EtOH is more lipophilic than MeOH and thus more harmful to the cell membranes (Patra *et al*. 2006). The final concentration of MeOH/EtOH in the 1–2% range is considered to be tolerated by most cells. Acetonitrile is also a common solvent for a range of chemicals. It has been suggested that the systemic toxic effects of acetonitrile are mainly mediated through the metabolic transformation into cyanide in the cell (Tanii and Hashimoto 1984). Like MeOH and EtOH, acetonitrile has high volatility and is shown to affect membrane systems (Yoshida *et al*. 2018). However, a final concentration of 0.3% is often considered safe for common cell culture systems.

**Figure 4.**
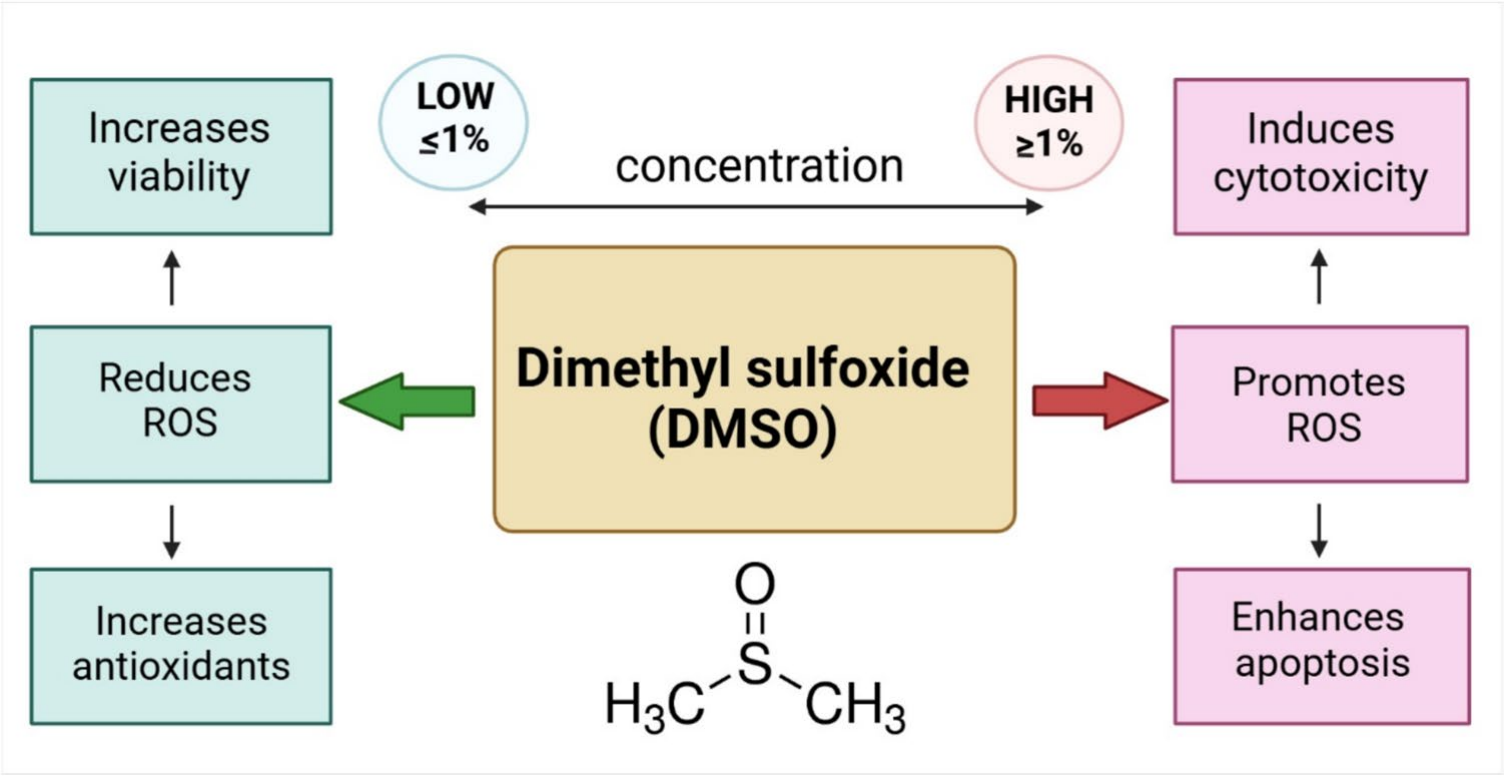
Cellular effects of DMSO regarding viability and ROS. The figure is modified from Dludla *et al*. (2021). Figure created in BioRender.com.

DMSO is a popular solvent for doing in vitro experiments. It is known to interact strongly with phospholipids, and then facilitate the uptake across biological membranes (Dludla *et al*. 2021), and it does not evaporate and is easy to pipette; however, there should be consideration of the overall percentage of DMSO. If the DMSO concentration is not only low, in the range of ≤ 1%, but also as low as < 0.1%, there is often observed a reduction in reactive oxygen species (ROS), an increase in cell viability, and an increase in antioxidant levels (glutathione, superoxide dismutase, catalase). In contrast, high DMSO concentrations, in the range of ≥ 1% are known to promote oxidative stress and enhance apoptosis and cytotoxicity (Dludla *et al*. 2021) (Fig. 4). Thus, it is recommended to use a final concentration of DMSO of 0.1–0.5% to ensure that the solvents minimally affect the cells. Overall, DMSO is often the choice of carrier for most experiments due to its stability at higher temperatures, high solubility for lipophilic compounds, easy handling, and low toxicity.

### Assay controls

When performing an experiment, it is important to include the correct controls. The assay should include a *negative control*, where cells are not exposed to any test compound, and should result in normal cell behaviour. You should also include a *positive control* that will give you information on assay performance. A *solvent control*, where the cells are exposed to the carrier solvent alone, is also needed. This gives you information on how the solvent alone affects your cells. When comparing the negative control with the solvent control effects of the solvent alone, such as changes in cell proliferation, cell morphology or cell death may be identified. It is crucial to keep the solvent concentrations constant across the dilution series of your test compound. However, it is important to be aware of the possibility of synergistic effects of the test compound together with the solvent, but this is difficult to elucidate as the test compound needs to be dissolved in a carrier solution. Additional controls for autofluorescence (Section “The exposure compound”) and staining specificity (isotype controls) should be considered.

### Dosing method

The method of adding the test compound to the cells in culture should be considered. There are two primary modes of delivery: direct and indirect. For direct exposure, the test compound is added directly to the wells containing cells and the exposure medium. In comparison, when the test compound is first diluted in a tube of exposure medium and then added to the well the mode of delivery is indirect. If using a direct exposure method, several 1000 × concentrated stock solutions of different concentrations of the toxicant can be made so the final concentration of solvent, such as DMSO, is 0.1% in your wells. Direct dosing is best suited for plates up to 48 wells, where the final volume of exposure medium is > 500 µL. It can be challenging to directly dose plates with 96 wells or greater due to the small volume of stock solution that needs to be added directly to the well. In addition, there may be varying results depending on whether the culture plate is gently agitated immediately after the introduction of the toxicant, or if the toxicant is instead allowed to passively diffuse in the exposure medium (Dayeh *et al*. 2025). Indirect dosing allows for a larger volume of toxicant-spiked exposure media to be introduced into the well in an easier and more precise fashion. However, for highly lipophilic compounds that stick onto plastic lab ware (Section “The exposure compound”, “Bioavailability”), it is advisable to use glass tubes when preparing the exposure solutions to avoid loss of your lipophilic exposure compound. In contrast to direct exposure, indirect exposure is compatible with 96- and 384-well tissue culture plates.

**Figure 5.**
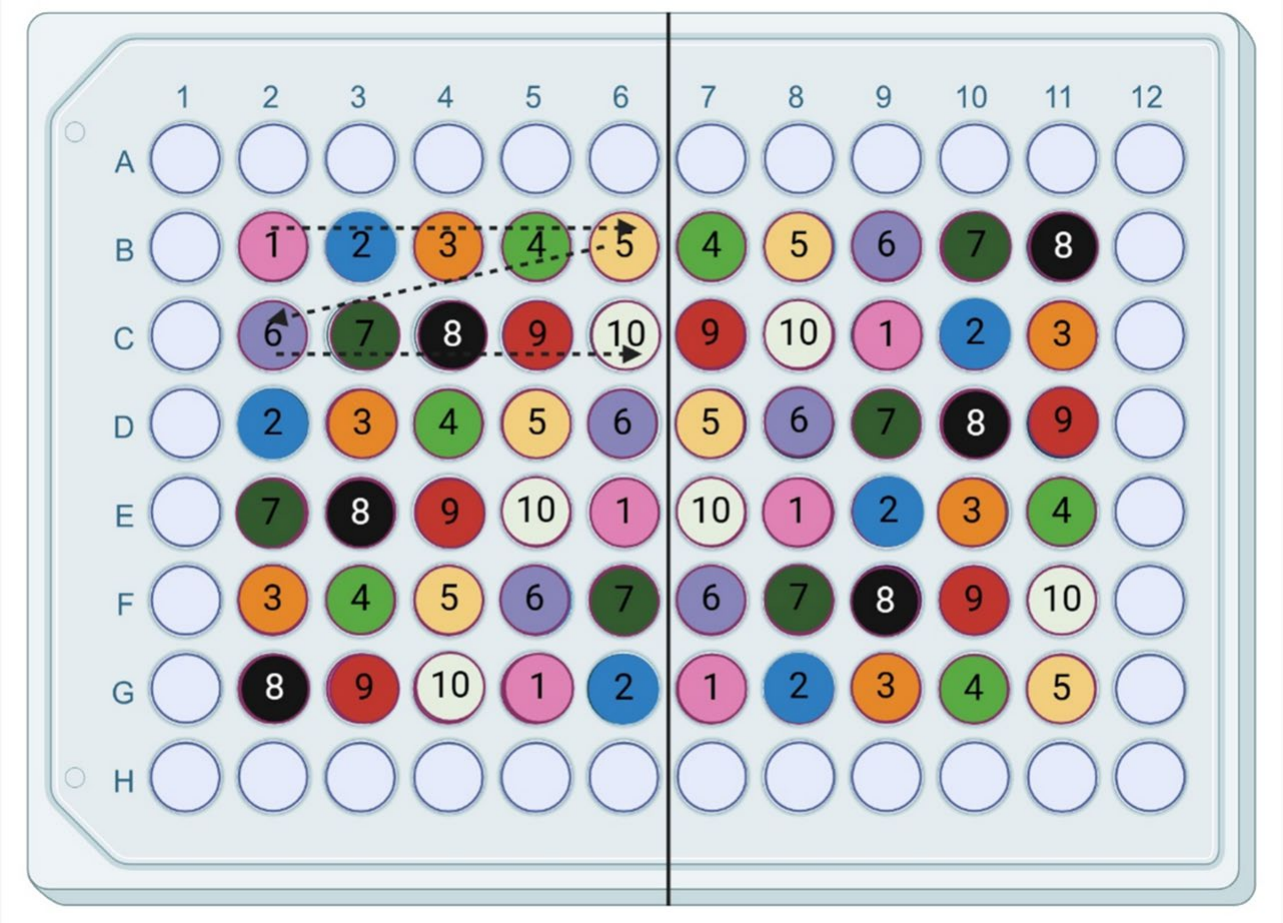
An example of randomized exposure, in a somewhat organized order in a 96-well plate; the example includes 10 independent samples with 6 technical parallels. The outer wells are without cells, filled with PBS to limit the edge effect. Created in Biorender.com.

Another consideration to avoid small differences over the 96-well plate is to do the exposure in a somewhat randomized order so the technical parallels are not close to each other (Fig. 5). It might be challenging to keep track of the samples, but as a minimum, several controls across the plate should be included. Also, by using bigger wells, such as 48-well plates instead of 96-well plates, a larger volume of cell culture medium and cell number makes it more robust due to smaller differences during pipetting.

### Common readouts – Microscopy – “seeing is believing”

It is recommended to visualise the cells using a microscope multiple times through the bioassay. The first observation should be before seeding the cells to ensure that the cells are healthy. Additional microscopic evaluations should occur immediately after seeding to determine consistency during the seeding process. The cells should be viewed just before exposure to the toxicant and immediately after exposure to note any changes in cell morphology due to the dosing methodology. A final microscopic evaluation of the cell culture should be done at the end of the exposure just before adding the cell viability fluorophores to determine if there are any toxic effects that are microscopically visualized.

### Viability assays

A combination of three viability assays: Alamar Blue, CFDA-AM, and Neutral Red cover a range of endpoints and are the basis of the OECD Test No. 249 (OECD 2021).

**Figure 6.**
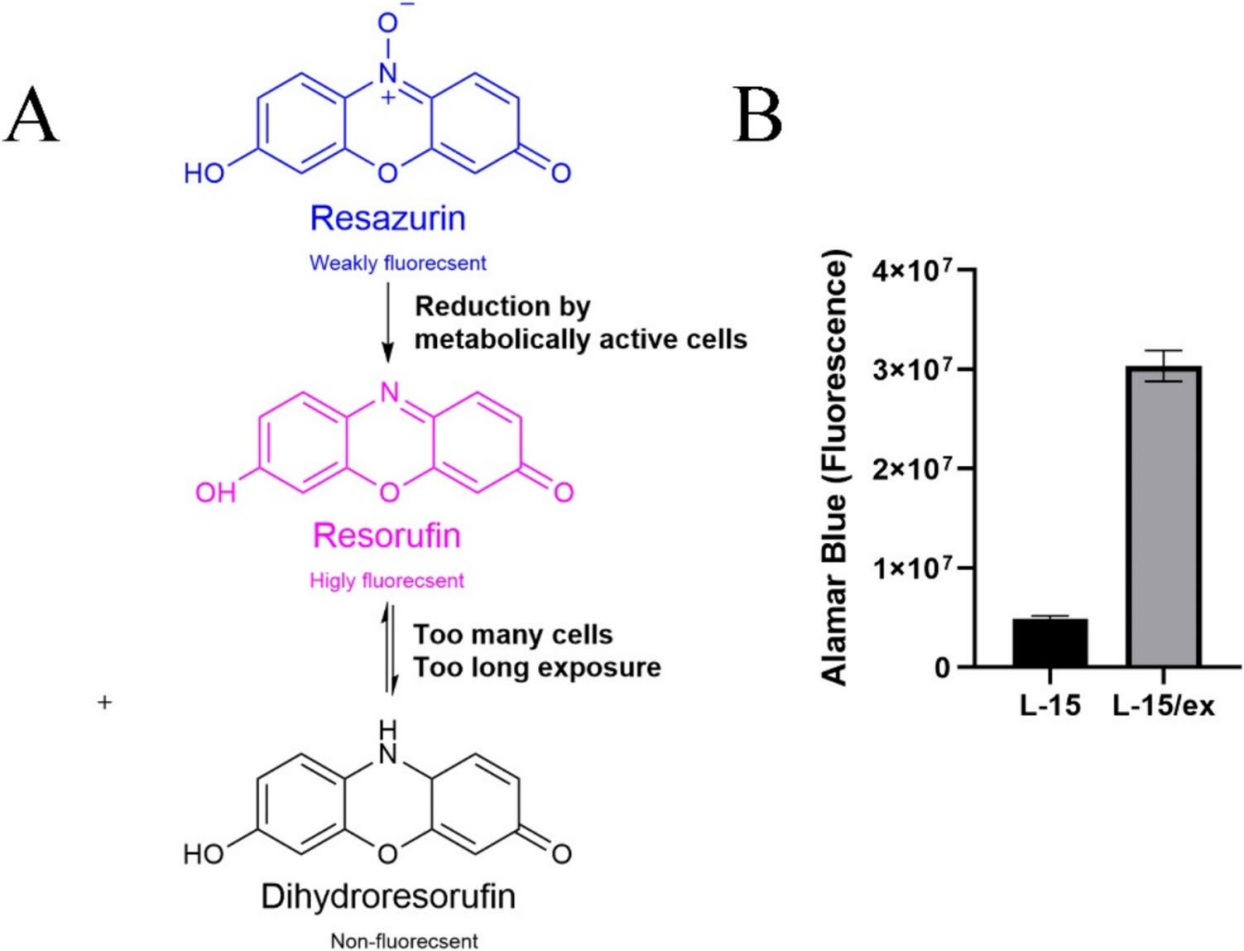
(*A*) Alamar Blue metabolism (ChemDraw). (*B*) Relative fluorescence of resorufin in fish cells (ASG- 10) incubated with Alamar Blue (5%) in combination with L- 15 or L- 15/ex medium for 2 h and resorufin fluorescence measured with a fluorometric plate reader.

Alamar Blue measures metabolic activity (O'Brien *et al*. 2000). The dye is non-toxic and is sensitive compared to other metabolic/mitochondrial assays such as MTT. Alamar Blue is a commercial preparation of rezasurin, which is dark blue in colour and is weakly fluorescent. The resazurin is reduced by metabolically active cells to resorufin, which is pink in colour and is highly fluorescent (Fig. 6*A*). Furthermore, if there are too many cells in the culture or if the dye incubation period goes beyond the recommended duration, the resulting accumulation of resorufin may undergo further reduction to the colourless, and non-fluorescent, compound dihydroresorufin (Fig. 6*A*) (Uzarski *et al*. 2017). Exposures with fish cells commonly use L- 15/ex medium, as described in the section “The exposure compound”, “Bioavailability”. However, it should be noted that the cells are more metabolically active in the L- 15/ex medium compared to the L- 15 medium (Fig. 6*B*). Consequently, only 1 h of incubation with Alamar Blue in the L- 15/ex medium is needed to give a sensitive readout. In contrast, 3–4 h is required when the incubation is done in the ordinary L- 15 medium. When working with Alamar Blue, one should be aware that stressed cells might exhibit an increase in metabolic activity above control levels (Solhaug *et al*. 2024a). It should also be noted that when the Alamar Blue stock gets old, it has a tendency to form small, barely visible precipitates that upon addition to the well make the cell culture medium dark blue which thus does not represent a good viability reading. However, this can be easily solved by sterile-filtering the Alamar Blue solution before use. In several applications, Alamar Blue may also be a better choice than the comparable MTT-assay, as Alamar Blue is non-toxic to the cells and the cells can be used for other applications after running the viability assay, such as replacing with culture medium for a recovery period (Dayeh *et al*. 2013). Additionally, substrates/inhibitors of efflux pumps interfere with MTT assay (Vellonen *et al*. 2004). As a consequence, if the MTT assay is used to quantify cell viability after exposure to toxins that also affect efflux pumps, such as the mycotoxin group Enniatins (Dornetshuber *et al*. 2009), it will give underestimated viability data.

The second OECD Test No. 249 viability assay measures cell membrane integrity using 5-carboxyfluorescein diacetate acetoxymethyl ester (CFDA-AM). This nonpolar, non-fluorescent dye rapidly diffuses into cells. If a cell has an intact membrane, the CFDA-AM is converted by nonspecific esterases into the polar and fluorescent dye, 5-carboxyfluorescein (CF) that diffuses out of cells slowly. This dye can be used in combination with Alamar Blue as the respective excitation and emission wavelengths do not overlap. Since the cell distribution in the well may differ and/or be uneven, it is an advantage to read multiple single points in each well, also called “well-scan”, which is available for several plate readers. In addition, the readout can be done by fluorescent microscopy. However, it is then very important to do the reading with the plate reader first as microscopy may point-bleach the fluorescence during microscopy evaluation if not a fully automatic microscopy imaging system is used.

The final OECD Test No. 249 cell viability assay is Neutral Red (NR), which is a measure of lysosomal activity as viable cells accumulate NR (3-amino- 7-dimethylamino- 2-methylphenazine hydrochloride) into the lysosomes. As cells begin to die, their ability to incorporate neutral red diminishes. Thus, loss of neutral red uptake corresponds to loss of cell viability. The NR dye could be added either before or after toxicant exposure. If the NR is added to cells prior to toxicant exposure, the endpoint would be the release of NR. However, if the NR dye is added after toxicant exposure, then dye uptake is the measured endpoint. As the assay involves fixation of the cells, the cells cannot be used for other applications later. NR uptake can be measured by both absorbance and fluorescence, but fluorescence is the most sensitive. During the fixation step, some cells lines have the tendency to peel off the well surface. So do remember to check your cells using microscopy at the end of this assay to make sure that your readout is due to the loss of staining and not just the loss of cells during the fixation step.

### Complex model systems

So far, the development of modern in vitro fish models is limited. The models should lead to a better understanding of basic fish physiology and enable predictions of chemical uptake and bioaccumulation in fish, a part that is essential for risk assessment. The making of such complex in vitro model systems often involves different three-dimensional (3D) cell cultures (e.g. spheroids), co-cultures of two or more cell types, and the formation of multicell, organ-like structures (organoids). Cell cultivation in microfluidic flow systems (organ-on-a-chip systems) mimics the circulation of blood, water, or intestinal fluid that generates a force called shear stress. Shear stress deeply influences cellular processes and the transport of nutrients, soluble compounds, drugs, and signalling molecules. The organ-on-a-chip systems may involve co-cultures of two or more cell types from the same organ that allow studies of organ-typical cell–cell communications, and biophysical and biochemical functions normally not possible in monocultures (Leung *et al*. 2022; Srivastava *et al*. 2024). The connection of two or more cultivation chambers, each containing organ-specific cell cultures, allows interaction studies between organs, such as gill-liver and gut-liver. Today, numerous companies, for example React4 life (Genova, Italy), Ibidi (Gräfelfing, Germany), and Elveflow (Paris, France), provide microfluidic flow systems with many choices regarding fluidic systems and chips.

Cultivation of non-differentiated fibroblasts from rainbow trout (*Oncorhynchus mykiss*; the RTG-2 cell line) as organoids produced a model that resembled more with the in vivo situation in *Saprolegnia* infection than traditional 2D cell cultures (Faber *et al*. 2021). Furthermore, liver spheroids were successfully made from the rainbow liver cell line RTL-W1 (Lammel *et al*. 2019; Holgersson *et al*. 2024) and primary liver cells (Baron *et al*. 2012; Hultman *et al*. 2019). Major benefits of using 3D spheroids are that their microenvironment has a high similarity to in vivo tissue and that considerably longer exposure times can be used. The cells also had increased metabolic capacities when organized as spheroids compared to conventional 2D cultures. Moreover, since liver spheroids stay metabolic active for a longer time, they are optimally suited for the determination of biotransformation pathways in vitro, especially with regard to slowly metabolizing substances that require long incubation times, which might exceed the lifetime of traditional hepatic metabolism assays using liver microsomes or S9 fractions (Hultman *et al*. 2019). A promising “fish gut-on-chip” microfluidic model was recently established by the research group of Kristin Schirmer (Drieschner *et al*. 2019). It allows investigating the intestinal barrier, leading to a better understanding of basic fish physiology, and makes it possible to predict chemical uptake and bioaccumulation in fish, which is essential for environmental risk assessment. The model is based on the rainbow trout cell lines, RTgutGC (intestinal epithelium) and RTgutF (intestine fibroblasts) grown in co-culture. Applications of the model have revealed that physiological, realistic fluid flow, and shear stress promoted a stable intestinal epithelial tightening compared to the static conditions in older test systems. Also, co-cultures of the Atlantic salmon epithelial gill cell line ASG-10 and the Atlantic salmon fibroblast cell line ASG-13 were found to increase the barrier, but did not decrease the permeability towards Lucifer yellow (Slattery *et al*. 2023). However, microfluidic flow has not been tested on this system yet.

When employing such complex in vitro systems, a comparison with a simpler system should be done to ensure that the results are realistic. If the results are the same, just use the simplest system. Furthermore, several microfluidic systems contain a lot of tubing and leakage is a common problem, so make sure that sealing tape is available. Furthermore, it is important to optimize the flow rate of the microfluidic system, as too high a flow may lead to detachment of the cells, but too low a flow may not give any difference compared to a static system. Also, if the exposure reagent is a lipophilic compound, be aware that a considerable amount of the compound might be attached to the plastic, as discussed in the section “The exposure compound,” “Bioavailability”.

## Summary

Overall, the creation of fish cell lines is a complex and labour-intensive process that requires a deep understanding of cell biology and techniques in cell culture. From primary fish cell culture origin to development of fish cell lines, and the application of fish cell lines in various areas, such as toxicology, the collective knowledge discussed in this paper should ideally enable future researchers to gain insight that it is the small things that matter.

## Data Availability

The raw data used are available from the corresponding author unpon resonable request.
